# A reverse genetic approach identifies an ancestral frameshift mutation in *RP1* causing recessive progressive retinal degeneration in European cattle breeds

**DOI:** 10.1186/s12711-016-0232-y

**Published:** 2016-08-10

**Authors:** Pauline Michot, Sabine Chahory, Andrew Marete, Cécile Grohs, Dimitri Dagios, Elise Donzel, Abdelhak Aboukadiri, Marie-Christine Deloche, Aurélie Allais-Bonnet, Matthieu Chambrial, Sarah Barbey, Lucie Genestout, Mekki Boussaha, Coralie Danchin-Burge, Sébastien Fritz, Didier Boichard, Aurélien Capitan

**Affiliations:** 1 UMR 1313 GABI, INRA, AgroParisTech, Université Paris-Saclay, 78350 Jouy-en-Josas, France; 2 ALLICE, 149 rue de Bercy, 75595 Paris Cedex 12, France; 3 Ecole Nationale Vétérinaire d’Alfort, Unité d’Ophtalmologie, Université Paris-Est, 7 avenue du Général de Gaulle, 94704 Maisons-Alfort Cedex, France; 4 Center for Quantitative Genetics and Genomics, Aarhus University, Aarhus, Denmark; 5 UMR 1198, Biologie du Développement et Reproduction, INRA, 78350 Jouy-en-Josas, France; 6 Origenplus, 38 rue de la Mérillière, 61300 L’Aigle, France; 7 UE 326, Domaine Expérimental du Pin-au-haras, INRA, 61310 Exmes, France; 8 Labogena DNA, Domaine de Vilvert, 78350 Jouy-en-Josas, France; 9 IDELE, 149 rue de Bercy, 75595 Paris Cedex 12, France

## Abstract

**Background:**

Domestication and artificial selection have resulted in strong genetic drift, relaxation of purifying selection and accumulation of deleterious mutations. As a consequence, bovine breeds experience regular outbreaks of recessive genetic defects which might represent only the tip of the iceberg since their detection depends on the observation of affected animals with distinctive symptoms. Thus, recessive mutations resulting in embryonic mortality or in non-specific symptoms are likely to be missed. The increasing availability of whole-genome sequences has opened new research avenues such as reverse genetics for their investigation. Our aim was to characterize the genetic load of 15 European breeds using data from the 1000 bull genomes consortium and prove that widespread harmful mutations remain to be detected.

**Results:**

We listed 2489 putative deleterious variants (in 1923 genes) segregating at a minimal frequency of 5 % in at least one of the breeds studied. Gene enrichment analysis showed major enrichment for genes related to nervous, visual and auditory systems, and moderate enrichment for genes related to cardiovascular and musculoskeletal systems. For verification purposes, we investigated the phenotypic consequences of a frameshift variant in the *retinitis pigmentosa*-*1* gene segregating in several breeds and at a high frequency (27 %) in Normande cattle. As described in certain human patients, clinical and histological examination revealed that this mutation causes progressive degeneration of photoreceptors leading to complete blindness in homozygotes. We established that the deleterious allele was even more frequent in the Normande breed before 1975 (>40 %) and has been progressively counter-selected likely because of its associated negative effect on udder morphology. Finally, using identity-by-descent analysis we demonstrated that this mutation resulted from a unique ancestral event that dates back to ~2800 to 4000 years.

**Conclusions:**

We provide a list of mutations that likely represent a substantial part of the genetic load of domestication in European cattle. We demonstrate that they accumulated non-randomly and that genes related to cognition and sensory functions are particularly affected. Finally, we describe an ancestral deleterious variant segregating in different breeds causing progressive retinal degeneration and irreversible blindness in adult animals.

**Electronic supplementary material:**

The online version of this article (doi:10.1186/s12711-016-0232-y) contains supplementary material, which is available to authorized users.

## Background

Domestication has had a dramatic effect on the genomes of plant and animal species. Reduction of environmental pressure combined with rapid growth of populations after strong demographic bottlenecks have resulted in relaxation of purifying selection and accumulation of deleterious mutations [1–5]. In the last 150 years, this phenomenon termed “the cost of domestication” has been particularly amplified in cattle because of the creation of breeds from a limited number of founder animals, overuse of a few elite sires with artificial insemination (AI) and intensive selection on specific traits. As a consequence most bovine breeds experience regular outbreaks of recessive genetic defects. With the advent of high-throughput genotyping and next-generation sequencing, efficient methods have been developed to identify the underlying mutations in record time and with a limited number of available cases [6, 7]. However, such approaches rely on the observation of affected animals with distinctive symptoms. It can be anticipated that the genetic defects reported so far represent only the tip of the iceberg and that many recessive mutations resulting in embryonic mortality or in non-specific symptoms, which can be confounded with those of common diseases, remain to be discovered. In addition to the influence of genetic drift and hitch-hiking, the frequency of some deleterious mutations, which would be detrimental in the wild, may have been involuntarily increased by artificial selection on behavior, coat color, morphological or production traits. This is the case for example for double-muscling, which causes dystocia [8, 9], and for a series of mutations under balancing selection [10, 11].

The increasing number of available whole-genome sequences (WGS) has recently opened new research avenues such as reverse genetics to investigate recessive defects. This strategy seems particularly suitable in cattle for which the sequencing of the most influential AI bulls of each breed (e.g. 1000 bull genomes project [12]) enables the identification of the vast majority of the non-private deleterious mutations that segregate in these populations. Furthermore, the inclusion of a subset of these polymorphisms into single nucleotide polymorphism (SNP) chips that are used for genomic selection should facilitate the detection of homozygotes (or of a deficit in homozygotes) for deleterious alleles among the tens of thousands of animals genotyped each year. In parallel, crossing genotyping data with pedigree information should enable the detection of severely affected homozygotes among animals that are born from at risk matings, which would not have been genotyped for genomic selection purposes. Finally, bovine populations provide an important number of cases available for sampling and experimental study to evaluate the functional consequences of the mutation, which is hardly possible in humans.

The purpose of this study was twofold: (i) to characterize the genetic load of 15 beef and dairy breeds using whole-genome sequencing data from the 1000 bull genomes consortium [12] and (ii) to prove that widespread harmful mutations remain to be detected in our cattle populations by characterizing the effect of a frameshift mutation in the *retinitis pigmentosa*-*1* (*RP1*) gene, which segregates in Normande cattle and other European breeds.

## Methods

### Ethical statement

Blood and ear biopsies were collected by veterinarians or by agricultural technicians licensed by the French Departmental Breeding Establishments [Etablissements Départementaux de l’Elevage (EDE)] during routine ear tagging, sampling for annual prophylaxis, paternity testing and genotyping for genetic defects or genomic selection. Ophthalmologic examinations and electroretinograms were approved after ethical evaluation by the ComERC committee (Ethical Committee for Clinical Research at the French Veterinary School of Maisons Alfort (ENVA) (Saisine n°14-01-2015) and performed under sedation controlled by a veterinarian specialized in cattle.

Invasive procedures were performed post-mortem after slaughter for meat production. Experiments reported in this work comply with the ethical guidelines of the French National Institute for Agricultural Research (INRA). All the samples and data analyzed were obtained with the permission of breeders, breeding organizations and research group providers.

### Animals

Details on animals used for each analysis are presented in Additional file 1: Table S1.

### Filtering of variants from whole-genome sequence data and prediction of their phenotypic consequences

Variants were selected from whole-genome sequence data of 1147 bulls from the 1000 bull genome project (for details on variant calling see Daetwyler et al. [12]). Briefly, raw reads were filtered and trimmed on chastity and quality score, then aligned on the UMD3.1 bovine reference sequence assembly using BWA [13]. SNPs and InDel were called from pooled bam files using SAMtools 0.1.18 mpileup [14]. Variants were then annotated using Ensembl Variant Effect Predictor [15]. Frequencies and allele counts were calculated across and within breeds using vcftools “freq” and “count” options [16]. Filtering consisted in selecting biallelic variants which (i) were predicted to cause a loss of protein function (i.e. affecting initiator codons, splice acceptor or donor sites, or causing a frameshift, a stop loss or gain, or a missense with a SIFT score of 0 [17]), (ii) had a calling quality (QUAL) above 30, (iii) presented a mapping quality (MQ) score of 59 or 60, (iv) had less than 5 % of animals with missing genotypes, and (v) had a minor allele frequency (MAF) higher than 5 % for at least one breed with a minimum of 20 individuals in the dataset (which means that alleles observed only once were not considered). It should be noted that variants with a SIFT score less than 0.05 are generally considered deleterious. In this study, we chose to retain only missense variants with a SIFT score of 0 to reduce possible artifacts. Furthermore, including missense deleterious variants with a SIFT score between 0.01 and 0.05 would have resulted in considering approximately one fourth of the total number of bovine genes, thus preventing subsequent gene enrichment analysis. In addition, each variant was manually checked to eliminate artifacts due to (i) adjacent substitutions within the same codon which are not accounted for in variant annotation, (ii) errors of annotations after comparing gene annotations from the UCSC and Ensembl genome browsers (http://genome.ucsc.edu, http://www.ensembl.org) annotations, (iii) repeated sequences (downloaded at http://genome.ucsc.edu, accession 21/10/2015). Only variants with a known official gene symbol were considered in the subsequent analyses.

To anticipate the phenotypic consequences of the mutations, annotations were completed by information on genetic syndromes associated with mutations within the same genes in humans (Online Mendelian Inheritance in Man, OMIM; http://www.omim.org) and mouse (Mammalian Phenotypes; http://www.informatics.jax.org) (see Additional file 2: Table S2).

### Gene set enrichment analysis

Gene enrichment analysis was performed using Ingenuity Pathway Analysis software (http://www.ingenuity.com/products/ipa/, [18]). We focused on “top canonical pathways” with a *p* value lower than 0.01 and “diseases and bio functions” annotations with a p value lower than 0.05. Annotations related to cancer and the general pathways entitled “skin lesion” and “liver lesion” were not considered since their results suffer from a bias. Pathways related to drug metabolism, which were not relevant for this study, were also eliminated. In addition, a unique keyword was assigned to each significantly enriched function annotation, with particular attention paid to the attribution of keywords related to subcellular portions, cell types and organs rather than to general processes. When possible, keywords appearing only once were regrouped with higher order items (e.g. cell type changed for organ, or process changed for the category defined by IPA) or with the predefined IPA “categories”. Frequency of keywords was used to set the size of the words in the word cloud representation.

### Ocular examination

Twenty-three pure and crossbred Normande cows from the INRA experimental facility of Le Pin-au-Haras (Normandy, France) with genotypes available for the frameshift mutation in the *retinitis pigmentosa*-*1* (*RP1*) gene were selected for ocular examination (see Additional file 1: Table S1). These consisted in four homozygous mutants, nine heterozygous and ten homozygous wild type animals. All these animals were in good health conditions and with no signs of systemic disease at the time of the study. Genotypes were not disclosed to the veterinarian to exclude any bias of personal interpretation. Examinations were performed indoors under ambient light. Visual performance was evaluated by the menace response test and dazzle and pupillary light reflexes (direct and indirect) were assessed with a Finoff transilluminator. Slit-lamp biomicroscopy (Kowa SL-15, Kowa Company) was performed before and after pupillary dilation using one drop of 1 % tropicamide. Fundi were examined by indirect ophthalmoscopy (Heine Omega 100, Heine Optotechnik, GmbH & CoKG) with 28-D and 20-D lenses.

### Electroretinogram tests

Electroretinogram tests were performed on two 5.5-years old cows, one homozygous wild type and one homozygous mutant, with a Retiport (Roland Consult, Brandenburg, Germany), under sedation (Xylazine 0.04 mg/kg IM) and after pupillary dilation (tropicamide eyedrops) and blocking of the auriculopalpebral nerve by subcutaneous injection of lidocaine. Topical tetracaïne eyedrops were used to anesthetize the ocular surfaces and corneas were lubricated by topical application of sodium hyaluronate 1.2 % during the test. The following responses were recorded: rod response before and after dark adaptation for 20 min, following a dim white stimulus (0.02–0.03 cd/m^2^/s), mixed response following four bright white flashes (2–3 cd/m^2^/s) at a rate of 0.1 Hz and cone response following four bright white flashes (2–3 cd/m^2^/s) at a rate of 5 Hz.

### Genotyping of Normande cattle that were reported to the French National Observatory of Bovine genetic abnormalities for progressive loss of vision

Twenty-eight Normande cows that were reported to the French National Observatory of Bovine genetic Abnormalities (ONAB) with signs of progressive loss of vision and blindness were genotyped for the *RP1* frameshift mutation. Genomic DNA was extracted from blood or ear biopsies using a standard phenol–chloroform protocol and genotyped by PCR and Sanger sequencing for the Chr14 g.23995411_23995412insA mutation. PCR primers were designed from the UMD3.1 bovine genome assembly with Primer3 software [19]) to span the insertion (left: TGCACAGGAAACCATATTGC and right: TTGCCCTAGTTGTGACATGC). Reactions were performed using the Go-Taq Flexi DNA Polymerase (Promega) according to the manufacturer’s instructions on a Mastercycler pro thermocycler (Eppendorf). The resulting amplicons were purified and bidirectionally sequenced by Eurofins MWG (Germany) using conventional Sanger sequencing. Polymorphisms were detected with the novoSNP software [20].

### Estimation of the allelic frequency of the *RP1* frameshift mutation

The *RP1* frameshift mutation was included in the Illumina EuroG10K custom SNP chip, which is routinely used for genomic selection in France. Thus, in addition to the 1000 genome dataset, genotypes for this mutation were available for 53,279 Holstein, 40,548 Montbéliarde, 12,106 Normande, 1634 Abondance, 1005 Red Pied Lowland, 698 Tarentaise, 579 Simmental, 507 Vosgienne, and 296 Brown Swiss animals.

### Post-mortem ocular examination and histological analysis

The eyes of two homozygous mutant cull cows (aged 8 years) that displayed a severe phenotype and two control cows (one 6-year-old heterozygous Normande and one 8-year-old homozygous wild-type Holstein) were collected post-mortem at the slaughterhouse (SVA Trémorel, France). One eye was dissected on site to perform a visual examination of the eye’s fundus and to collect the retina and choroid. Samples were immediately frozen in liquid nitrogen and stored at –80 °C until DNA extraction. The second eye was injected with 2.5 ml formaldehyde and fixed by a 24-h incubation in the same solution. The retina and choroid were subsequently dehydrated in a graded ethanol series, cleared with xylene and embedded in paraffin. Microtome sections. (5 µm, Leica RM2245) were stained with haematoxylin, eosin and saffron (HES). Digital images were obtained with the NanoZoomer 2.0- HT slide scanner (Hamamatzu).

### Association with recorded traits

At the time of the analysis, Illumina EuroG10K SNP chip genotype data were available for 7439 Normande animals, which had their sire genotyped with the Illumina BovineSNP50 chip. The Illumina EuroG10K SNP chip comprises the *RP1* frameshift mutation as well as more than 10,000 common SNPs with the Illumina BovineSNP50 chip. Using Fimpute [21] we were able to attribute a genotype for the *RP1* polymorphism to 48,715 additional animals that were previously genotyped with the BovineSNP50 chip. The complete dataset comprised 11,986 Normande cows with phenotype information on three coat colour phenotypes (proportion of white areas on the body; proportion of white areas on the face, and brindling intensity) and on 28 traits that are routinely recorded for genetic evaluations (milk yield, fat content, protein content, fat percent, protein percent, cell score, clinical mastitis, milking speed, stature, chest width, body depth, width at pin bone, rump angle, rear legs side view, rear legs rear view, back muscle, fillet muscle, rear muscle, fore udder attachment, rear udder height, udder balance, teat orientation, front teat distance, udder support, udder depth development, interval between calving and first insemination, fertility at insemination of lactating cows, fertility at insemination of heifers). Associations between the *RP1* frameshift polymorphism and traits were tested using GCTA [22]. Phenotypes were adjusted for environmental effects which were estimated in the national genetic evaluation procedure and assumed to reflect the genetic effect of the animal and a random residual effect. Therefore, the analysis model included only an overall mean, a polygenic effect, the effect of the genotype at the *RP1* frameshift polymorphism, and a residual. The polygenic effect was estimated by using a genomic relationship matrix that was derived from 43,801 SNPs on the Illumina BovineSNP50 chip. Finally, a Bonferroni correction that consisted in dividing the p value by the total number of tests performed was applied to account for multiple-testing.

### Across-breed identity-by-descent analysis around the *RP1* frameshift mutation

Identity-by-descent (IBD) analysis was performed to (i) test for the existence of one versus multiple mutation events in the different breeds and (ii) estimate the date of the origin of the mutation(s). For that purpose, phased genotypes for a 1.3-Mb region (Chr14:23474270-24643266; corresponding to the smallest IBD homozygous region detected in the genome of one homozygous mutant Normande AI bull, named Diametre (FR5388012666) were extracted for 35 heterozygous and three homozygous carrier animals identified among the 1147 animals from run 4 of the 1000 bull genomes project. Phasing was performed within the framework of the 1000 bull genome project using BEAGLE [12, 23].

Within this homozygous region of Diametre’s genome, 9448 SNPs with the highest quality score (QUAL = 999) were selected and considered as reference haplotypes. For each animal, the rate of homozygous genotypes in opposition with the chosen reference genotypes was calculated for sliding windows of 100 SNPs. Then, the number of individuals that had at least 5 % of inconsistencies with the reference haplotype was counted and attributed to the position of the 51th SNP in each window. This level of 5 % of inconsistencies was chosen to account for the low sequence coverage of certain animals and for the putative occurrence of de novo mutations over time in the vicinity of the old frameshift mutation. The IBD block around the frameshift mutation was finally defined by windows for which none of the carriers displayed 5 % or more of inconsistencies with the haplotypes of Diametre. For control purposes, the same process was applied to a set of 38 non-carrier animals that were randomly selected among individuals belonging to the same breeds as the carriers.

### Estimation of the age of the *RP1* frameshift mutation according to the size of the IBD segment shared among breeds

We considered that two animals that shared an IBD segment of size c (c being the size in Morgan) inherited this segment from a common ancestor that lived 1/(2 c) generations ago. We assumed that, on average, 1 cM corresponds to 1,000,000 bp and that generation intervals range from 5 to 7 years, depending on the breeding system (natural mating population or modern breeding schemes).

### Analysis of the changes in frequency of the *RP1* frameshift mutation in the Normande breed

To study the changes in allelic frequency of the *RP1* frameshift mutation in the Normande breed, first we developed a haplotype test using 15,515 animals (1077 homozygous carrier, 6363 heterozygous and 8075 homozygous wild type animals) that were genotyped for this variant with the Illumina EuroG10K custom SNP chip and had been phased and imputed for the Illumina BovineSNP50 markers within the framework of the French genomic selection [24]. The haplotype was fixed to 50 SNPs between SNPs ARS-BFGL-BAC-12159 (Chr14 position 22587081 bp) and ARS-BFGL-NGS-36089 (Chr14 position 25698286). We identified 691 haplotypes among which 12.45 % were associated to the frameshift mutation, 83.79 % were not associated with it and 3.76 % were classified as undetermined (i.e. detected in both homozygous carriers and non-carriers). When applied to all the Normande cattle phased Illumina BovineSNP50 genotyped data, 97.3 % of the haplotypes were assigned a status (27.26 % were associated to the frameshift mutation, 83.79 % were associated to the wild type allele, 1.03 % undetermined) and 2.47 % were classified as not documented due to lack of haplotype information among the animals genotyped with the EuroG10K chip. From these haplotype-allele associations, we estimated the genotypes for 1375 phased Normand AI bulls (born between 1975 and 2015) for the *RP1* frameshift polymorphism. Allelic frequencies were calculated over time for sliding windows of 7 years (i.e. on average one generation) after removing haplotypes without information.

## Results and discussion

### During domestication, deleterious mutations have accumulated in non random sets of genes

**Fig. 1:**
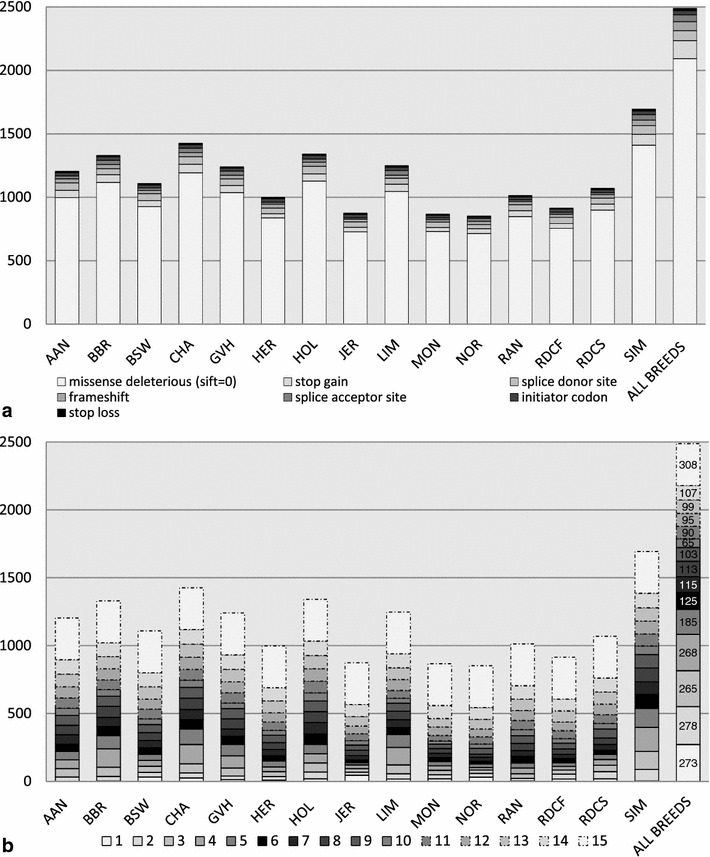
Details on non-rare putative deleterious variants selected in 15 breeds from the 1000 bull genomes run4 dataset. **a** Distribution of the variants by breed and type of mutations. **b** Distribution for each breed of the number of variants shared with other breeds. Note that only 18.3 % (273/2489) of non-rare putative deleterious variants are breed-specific. *AAN* Aberdeen-Angus, *BBR* Beef Booster Composite, *BSW* Brown Swiss, *CHA* charolais, *GVH* Gelbvieh, *HER* Hereford, *HOL* Holstein, *JER* Jersey, *LIM* Limousine, *MON* Montbéliarde, *NOR* Normande, *RAN* Red Angus, *RDCF* Finnish Red, *RDCS* Swedish Red, *SIM* Simmental

A series of filters was applied to draw a list of non-rare putative deleterious polymorphisms in the most important cattle breeds and to reduce as much as possible the false discovery rate (see “Methods” section). Since this study focused on non-rare variants, putative deleterious polymorphisms with a frequency lower than 5 % in all breeds were not investigated. This analysis yielded 2489 putative deleterious variants (stop lost and gained, frameshift, splice acceptor and donor sites, initiator codon variants and missense variants predicted as deleterious with a score of 0 by SIFT) that segregated at a frequency of 5 % or more in at least one of the 15 breeds represented by at least 20 genomes in run 4 of the 1000 bull genomes project [12] (Fig. 1; for details see “Methods” section). The distribution of these variants was similar in terms of number and type of mutations between breeds in spite of quite different numbers of sequenced animals. This result can be explained by the rather high variant frequencies considered. Interestingly, 89 % (2216/2489) of these polymorphisms were observed in more than one breed and as much as 12 % (308/2489) in all 15 breeds, which indicates (subject to any unregistered crossbreeding event) that the majority of the retained variants existed prior to the splitting of the different cattle populations studied (i.e. at least 500 years ago [25]).

A total of 1923 genes carried a deleterious mutation of which 566 counted two or more. A screening of phenotype databases revealed that 908 genes (1144 variants) were associated to at least one mammalian phenotype in laboratory animals (MGI database) and 375 (corresponding to 395 variants) with an inherited syndrome in humans (OMIM database). From our own interpretation, almost two-thirds of these syndromes described in mouse and humans presented a phenotype that would have been difficult to detect by the different national observatories for genetic defects in cattle (i.e. those affecting metabolism, immunity, cognition) (see Additional file 2: Table S2).

In this selection, we also retrieved five variants that were previously reported to cause major phenotypes in cattle. These comprise mutations that have been favored by artificial selection (i.e. p.Q204X mutation in *MSTN* for double muscling in Charolais [26]), or with a severe but invisible phenotype (i.e. p.R12X and p.R55X nonsense mutations in *SCL37A2* and *CWC15* for embryonic mortality in Montbeliarde [27] and Jersey [28], respectively), or with a mild phenotype that is present in several breeds (i.e. p.R238X mutation in *FMO3* for trimethylaminuria or “fishy-off flavor” of milk [29] and p.W80X mutation in *BCO2* for the “yellow color” of milk and fat [30]). These examples validate that such variants which are deleterious to the protein function may exist and segregate at moderate to high frequencies in cattle breeds.

**Fig. 2:**
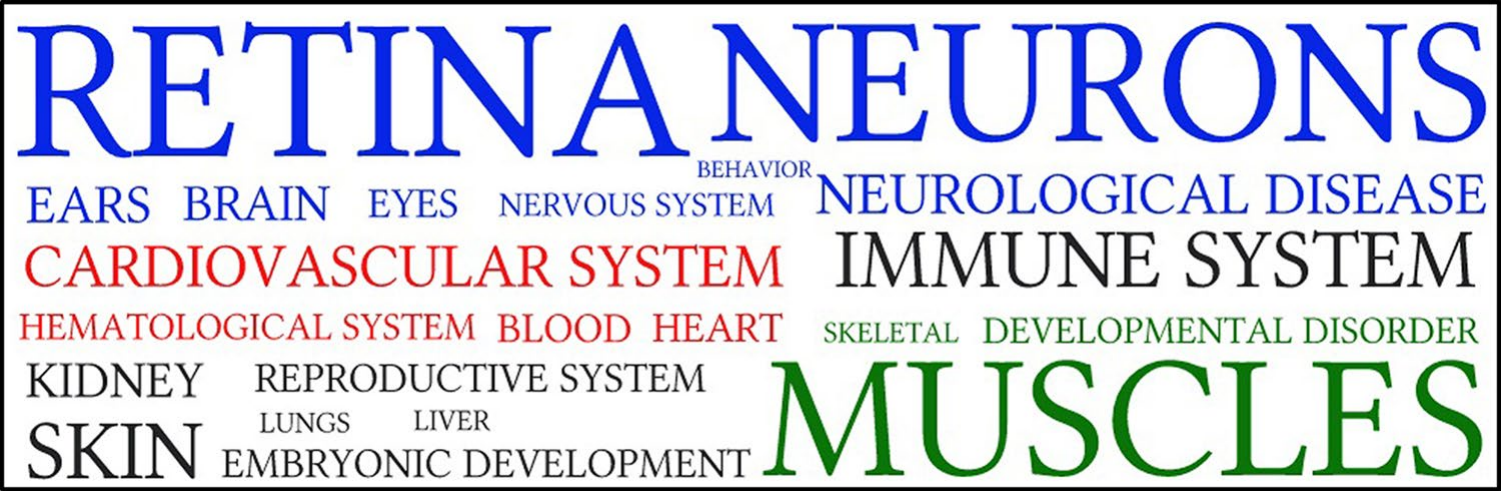
Word cloud representing the frequency of key-words assigned to significant ingenuity pathway analysis annotations. Only IPA annotations for “top diseases and bio functions” with a p value <0.01 were considered. The size of the font used is proportional to the frequency of each keyword associated with functional annotations. Keywords were clustered into overall related systems: (i) *blue* nervous, visual and auditory systems; (ii) *red* cardiovascular system; and (iii) *green* musculoskeletal systems. They represented respectively 41.5, 12.8 and 12.3 % of the functional annotations considered in the analysis. Annotations related to the two most frequent words, retina and neurons represent, respectively 10.8 and 8.7 % of the total number of annotations

To obtain an overall picture of the developmental pathways that are affected by our set of variants, we performed a gene enrichment analysis using the ingenuity pathway analysis (IPA) software [18]. This revealed an important enrichment for genes related to nervous system development and function and moderate enrichments for a limited number of other diseases, physiological and biological annotations (see Additional file 3: Tables S3, S4, and S5). We then analyzed the frequency of the keywords that were assigned to each annotation to gain further insight into the organs, tissues or systems represented (Fig. 2) and Additional file 3: Table S6. With 41.5 % of the word counts, the largest cluster was by far composed of words related to nervous, visual and auditory systems, which comprised genes involved in sensorial functions and/or cognition. Indeed, we noted as much as 17.7 % (72/407) of genes related to retina development and function, as well as genes involved in other defects of eye development such as cataract and microphtalmia, and genes associated with deafness (e.g. genes coding for cochlin, *COCH*; otogelin, *OTOG*; otogelin-like, *OTGL*; myosin heavy chain 15, *MYO15A*; and stereocilin, *STRC*) [31–35]. Note that we also detected a number of deleterious mutations in olfactory receptor genes which are not considered by IPA and thus were not accounted for in our analysis.

In addition, we retrieved important genes for neuro-cognitive functions which are associated with behavioral disorders in humans such as mental retardation, schizophrenia, bipolar disorder or autism. Among other examples, we can cite genes coding for glutamate receptors (*GRIK2* and *GRM7*), glutamate being the most important neurotransmitter in the brain, semaphorins which are involved, among other functions, in axon guidance (*SEMA3A*, *SEMA3B*, *SEMA4A*, *SEMA4D* and *SEMA5A*), calcium voltage channel subunits (*CACNA1C* and *CACNB2*), a receptor for neuroregulin 1 (*ERBB4*), the neurexin-3-alpha protein which has an important role in neuronal function (*NRXN3*), and a post synaptic protein (*SYNGAP1*) [36–45]. Interestingly, only four of the 407 genes from this cluster co-localized with selective sweeps in cattle, chicken, rabbit and/or pig (i.e. *GRIK2* in rabbit, *SEMA3* in pig and chicken, and *ERBB4* and *CACNA1C* in cattle) [46–49].

Therefore, whereas genes that are involved in sensory functions and cognition represent obvious targets of domestication [5, 46], it is unlikely that the variants reported here were positively selected during domestication or subsequent selection processes. More likely, our results indicate that, in a domestic context, such mutations were more tolerated than mutations affecting other systems which are of primary importance for production, reproduction and survival.

Analyzing the frequency of the keywords that were assigned to each IPA annotation also revealed two additional clusters related to cardiovascular (12.8 %), and muscle and skeletal systems (12.3 %), which might be associated with positive selection. These two clusters comprised genes that are associated with selective sweeps and/or production traits such as *MSTN* for double-muscling, *CCNL1* for reduced birth weight, *THADA* for body weight variation, *GOLGA4* for stature, and *LCORL* for stature and skeletal development in cattle [49–51] and *FANCA* for the cardiac system and *NR3C2* for blood pressure in horse [5]. Note that neuro-muscular interactions were also underlined by two IPA canonical pathways (p value <0.01), i.e. the “Agrin interactions at neuromuscular junctions” and “nNOS signaling in skeletal muscles”, which are both involved in neuro-muscular diseases and muscle dystrophies. Finally, two other canonical pathways, the “complement system” (part of the innate immune system of an organism) pathways and the “t-RNA charging” (regrouping the key enzymes of protein translation mechanisms in mitochondria) are relevant because of their involvement in two functions that are subject to important selection pressure i.e. immunity and energetic metabolism via mitochondrial function [52, 53] (see Additional file 3: Table S7).

### A frameshift mutation in the *RP1* gene causes progressive blindness in cattle

#### A good candidate for phenotype characterization

For validation, we decided to evaluate the phenotypic consequences of one mutation which was both (i) observed in numerous breeds and (ii) predicted to affect the organ that was most represented in the previous analyses, i.e. the retina. We selected a one base pair insertion (Chr14: g.23995411_23995412insA) that affects the *retinitis pigmentosa*-*1* gene (*RP1*) which segregates at a particularly high frequency in Normande dairy cattle (Table 1). This mutation is predicted to cause a frameshift at codon 791 and to terminate the protein 13 amino acids later (p. R791KfsX13). If synthesized, the resulting protein would be truncated at 40 % of its normal length and consequently lack two-thirds of its *C*-terminal end.

**Table 1:** Frequency of the *RP1* frameshift allele among breeds represented in run4 of the 1000 bull genomes project

Breed	Frequency of the *RP1* frameshift allele in % (number of genomes per breed with available genotype)
Aberdeen Angus	1.8 (140)
Beef Booster Composite	2.1 (2)
Belgian Blue	5.0 (10)
Charolais	3.8 (39)
Gelbvieh	1.4 (36)
Holstein	1.8 (312)
Maine-Anjou	14.3 (7)
Normande	28.3 (23)
Red Angus	7.0 (28)
Run4	1.8 (1137)

The *RP1* frameshift allele was absent from the Brown Swiss (n = 59), Finnish red (n = 25), Hereford (n = 34), Jersey (n = 60), Limousine (n = 33), Montbéliarde (n = 28), Simmental (n = 215) and Swedish Red (n = 31) breeds, which each totalized more than 20 animals in run4 of the 1000 bull genomes project and from 12 additional breeds, which each totalized less than 20 animals

In humans and mouse, similar truncation mutations in the *RP1* gene, which encodes a microtubule associated protein that is essential for the organization of the outer segments of the photoreceptors in the retina, have been reported to cause autosomal dominant and recessive retinitis pigmentosa [54–57]. Retinitis pigmentosa is a form of inherited degenerative retinal disorder that is characterized by progressive death of photoreceptor cells. Symptoms typically start with loss of night vision due to degeneration of rod-photoreceptors, followed by degeneration of cone-photoreceptors leading to loss of central vision and eventually to complete blindness [58].

For decades, Normande cattle have been considered to have poor eyesight, with older animals showing a typical loss of night vision or blindness. Because it was considered as a breed-specific trait, only a few cases had been reported to the French National Observatory of Bovine genetic Abnormalities (ONAB) and no genetic studies had been initiated. In a first attempt, we genotyped by PCR and Sanger sequencing 28 Normande cows that had been declared to the ONAB for partial or total blindness with no other indication of external eye affection. We observed a significant increase in the number of homozygous mutants (Chi square; p value = 0.003) in this group compared to the population of sequenced Normande founder sires (Table 2), which suggests that this frameshift mutation is responsible for a non-negligible part of the loss-of-vision problems observed in Normande cattle. As a consequence, we decided to include this variant in the EuroG10K SNP chip, to collect genotype information on the French bovine population that is genotyped for genomic selection and to identify carriers for subsequent phenotype characterization.

**Table 2:** Genotype frequencies for the *RP1* frameshift variant among 23 Normande founder bulls and 28 animals reported to ONAB for loss of vision

Genotype frequencies	Normande bulls in the 1000 bull genomes dataset (n = 23)	Loss of vision phenotype (n = 28)
Fs/Fs	8.7 % (n = 2)	50.0 % (n = 14)
Fs/Wt	43.5 % (n = 10)	35.7 % (n = 10)
Wt/Wt	47.8 % (n = 11)	14.3 % (n = 4)

Genotype frequencies calculated from the 23 Normande bulls available in run4 of the 1000 bull genomes project and from 28 cows declared to ONAB for loss of vision

*ONAB* French National Observatory of Bovine genetic Abnormalities, *Fs* frameshift allele, *Wt* wild type allele

The number of homozygous carriers (Fs/Fs) among affected animals is significantly larger than among the sequenced bulls (Chi^2^-test p value = 0.00226)

#### Clinical and histological tests revealed symptoms of retinal degeneration in homozygous mutants

**Fig. 3:**
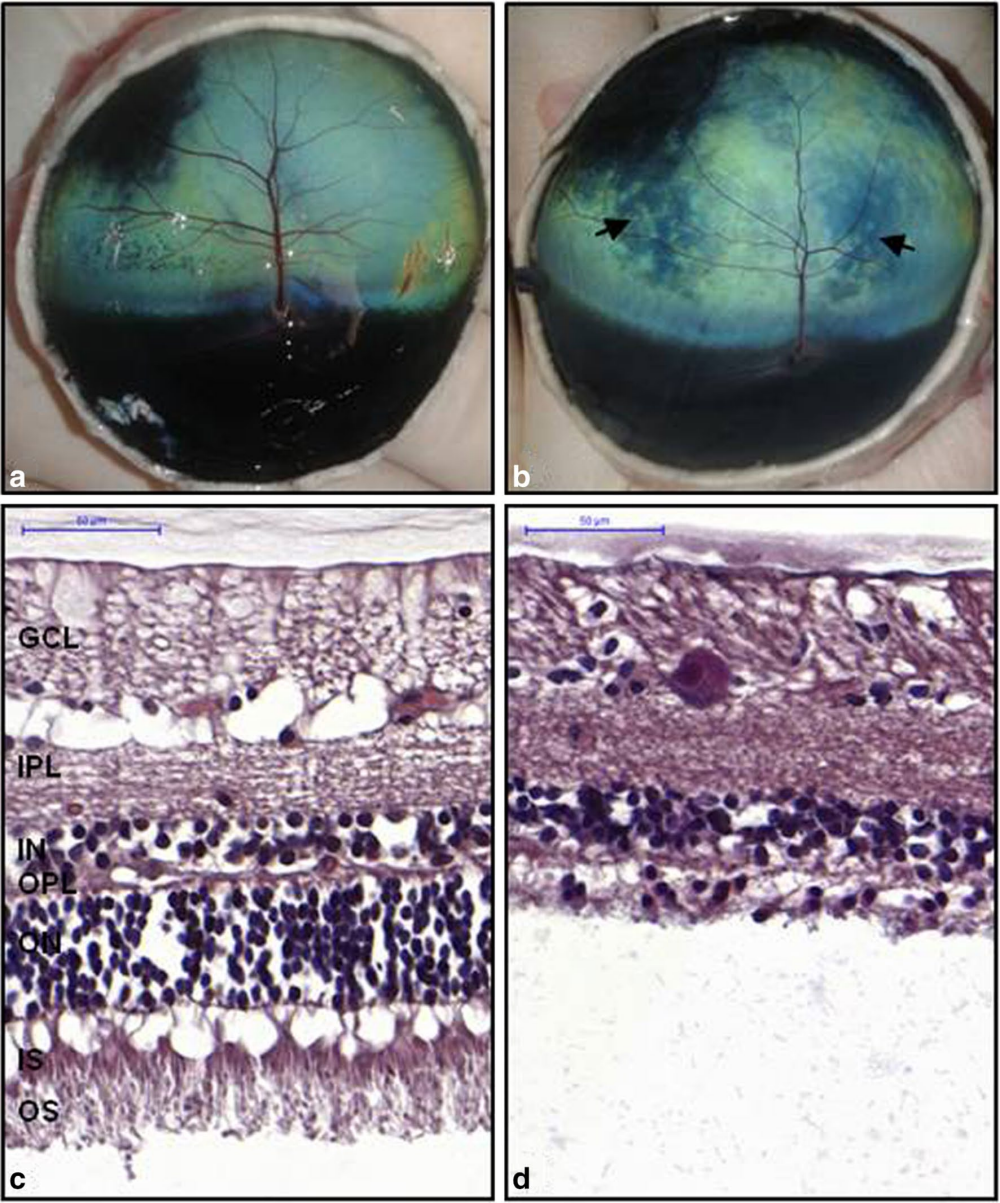
Clinical and histological features of retinal degeneration in old Normande cows. **a** and **b** Eyes fundus from control *RP1* Fs/Wt (**a**) and affected *RP1* Fs/Fs (**b**) Normande cows. *Arrows* indicate hyperreflective areas and note the general reduction of the caliber of blood vessels in the affected animal. **c** and **d** Histological sections of the retina from the same control (**c**) and affected (**d**) animals. Note the total absence of inner and outer segments of photoreceptor cells and a marked thinning and disorganization of the outer nuclear layer confirming retinal degeneration in the Fs/Fs animal. (*GCL* ganglionic cells layer, *IPL* inner plexiform layer, *INL* inner nuclear layer, *OPL* outer plexiform layer, *ONL* outer nuclear layer, *IS* inner segment, *OS* outer segment. 5 µm sections of tissue embedded in paraffin and stained with HES. The choroid is not shown due to large artefactual gaps created by tissue preparation)

To gain better insight into the phenotypic consequences of this frameshift variant, we performed ocular tests on 23 pure and crossbred Normande cows of the same herd and for which genotype information was available. Genotypes were not disclosed to the veterinarian to exclude any bias of personal interpretation. All heterozygous and homozygous wild-type animals showed normal vision. Only a small proportion of them (three homozygous wild-type and two heterozygous) presented uni- or bilateral focal hyper-reflective areas in the tapetal fundus, which had no apparent consequences on their visual acuity. Among the four homozygous mutant animals, two heifers aged less than 3 years had normal vision and ocular tests. In contrast, two older animals aged 4.5 and 5.5 years presented respectively marked visual deficit and blindness, in spite of normal pupillary light reflexes. Their ocular fundi showed typical features of bilateral retinal degeneration with a heterogeneous color, multiple focal areas of hyper reflectivity in the tapetal area which could be coalescent, and a reduction in the caliber of retinal blood vessels (Fig. 3; Table 3). Thus, their phenotype was clearly distinct from the three homozygous wild-type individuals and the two heterozygous animals that displayed minor abnormalities of the ocular fundus.

**Table 3:** Results of the ocular tests for the Normande dairy cattle examined

	Fs/Fs	Fs/Wt	Wt/Wt
Normal vision and eyes fundus	2 (<2 years)	7 (6.1 years)	7 (5.7 years)
Mild unilateral or bilateral focal retinal degeneration with preserved vision		2 (5.8 years)	3 (7.2 years)
Bilateral retinal degeneration with marked visual deficit	1 (4.5 years)		
Bilateral retinal degeneration with blindness	1 (5.5 years)		
Number of animals	4	9	10

Note that the pupillary light reflex was preserved in all the animals studied. The number of animals for each group and their average age in years (y) are presented

*Fs* frameshift allele, *Wt* wild type allele

Electroretinogram (ERG) performed on the oldest homozygous mutant confirmed the impairment of its retinal function with a lack of scotopic response and a reduced photopic response as compared with a wild-type control of the same age (Table 4).

**Table 4:** Electroretinogram results i.e. values of the amplitudes and the culminating times of a- and b-waves in one control and one affected animal

	Amplitude (μV)		Culminating time (msec)	
	a-wave	b-wave	a-wave	b-wave
Rod response				
Control	–	429	–	52
Affected	–	–	–	–
Mixed response				
Control	80.9	274	17	60
Affected	–	–	–	–
Cone response				
Control	36.7	275	13	24
Affected	15.6	43.6	17	27

Finally, to characterize this phenotype at the tissue level, we collected the retinas of two additional homozygous carriers (aged 8 years) and two control cows (one 6-year-old heterozygous Normande and one 8-year-old homozygous wild-type Holstein) after slaughter (Fig. 3). In concordance with previous analyses on the eyes’ fundus and retinal function, histological analyses revealed a total absence of photoreceptor outer segments along with a marked thinning and disorganization of the outer nuclear layer with very few remaining nuclei.

Taken together, these results provide strong support that the *RP1* frameshift mutation causes a recessive loss of vision in bovine cattle. The phenotype observed is similar to the description in humans with a late onset of the disease due to progressive degeneration of the photoreceptors. Very few genetic conditions that affect eyesight have been reported in cattle [59] and, to our knowledge, this is the first time that a mutation causing retinal degeneration is reported in this species. Indeed, while, in the past, several cases of progressive retinal degeneration were reported in Holstein cows, their genetic etiology has not been confirmed so far [60, 61].

### IBD analysis reveals a unique and ancestral mutation event

**Fig. 4:**
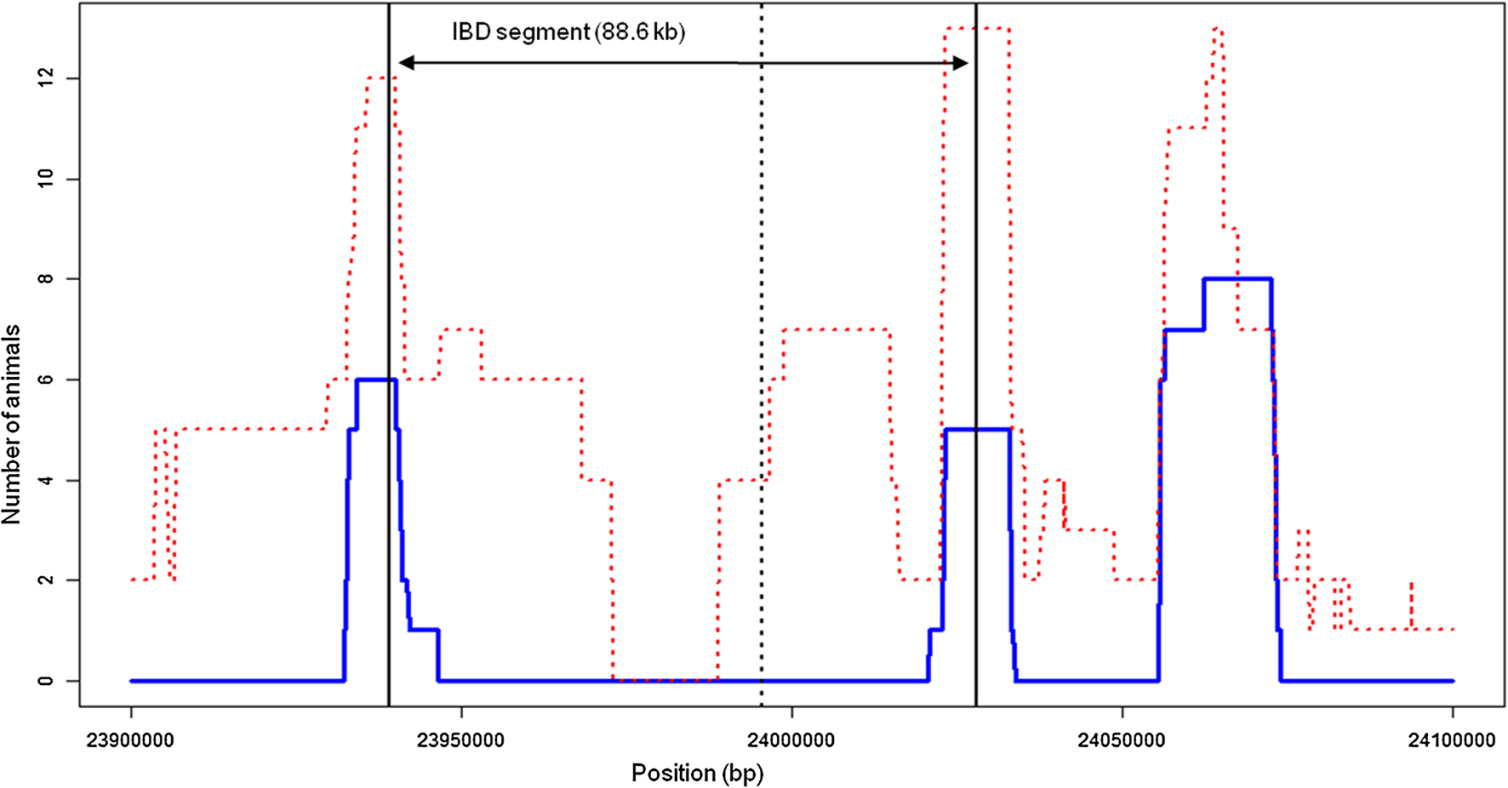
IBD analysis around the *RP1* frameshift mutation. *Blue line* number of animals carrying the *RP1* frameshift allele but showing at least 5 % of homozygous genotypes for the alternative allele as compared with the *RP1* Fs/Fs Normande bull Diametre in sliding windows of 100 SNPs. Thirty-five Fs/Wt and three Fs/Fs animals were considered. Successive windows around the mutation for which this count is null defines an 88.6-kb IBD segment common to all carriers. The same calculation was applied to a control group of 38 randomly chosen non-carrier bulls from the same breeds (*red dashed line*). Note that the segment around the mutation is not conserved in this group. The *black dashed* line indicates the localization of the mutation

As previously mentioned, the frameshift mutation in *RP1* is not restricted to the Normande breed. So far, we have identified carriers in at least 12 cattle breeds: nine of the 15 breeds from the 1000 bull genomes dataset used in this study (Holstein, Charolais, Normande, Red Angus, Aberdeen-Angus, Gelbvieh, Beef Booster Composite, Maine-Anjou and Belgian Blue), and the Montbeliarde, Abondance and Vosgienne breeds based on EuroG10K genotyping results (Table 5). Since the mutation consists in the insertion of one adenosine in a polynucleotide stretch, which is more prone to mutation than other sites of the genome, we performed an IBD analysis to verify if only one ancestral mutation or multiple independent mutation events accounted for the wide distribution of this variant (see “Methods” section). In the 1000 bull genomes dataset, we identified a unique fragment of 88.6 kb (Chr14:23939194-24027957) that encompasses the mutation (Fig. 4) and was shared by all carriers (N = 38) but absent in non-carriers from the same breeds. This confirms the existence of a unique ancestral mutation event which, according to the size of the IBD segment, was dated back to approximately 565 generations, i.e. 2800 to 4000 years before present, considering that the generation interval can vary from 5 to 7 years (see “Methods” section).

**Table 5:** Genotype frequencies for the *RP1* frameshift polymorphism from the EuroG10K genotyping results

Breed	Wt/Wt	Fs/Wt	Fs/Fs	MAF (%)
Abondance	1633	1	0	0.03
Brown Swiss	296	0	0	0.00
Tarentaise	698	0	0	0.00
Simmental	579	0	0	0.00
Montbéliarde	40,188	359	1	0.45
Normande	6294	4915	897	27.71
Vosgienne	505	2	0	0.20
Holstein	51,640	1627	12	1.55
Red pied lowland	1005	0	0	0.00

*Fs* frameshift allele, *Wt* wild type allele

The observation of this old variant at low to moderate frequencies in numerous bovine breeds could be explained by a combination of genetic drift and absence of or a very limited negative counter-selection due to the late onset of the defect. Nevertheless, the high frequency of this mutation observed in Normande cattle (27.7 % in the genotyped population for genomic selection) was particularly striking and led us to perform additional investigations to test for positive selection (either directly or mediated by hitch-hiking) in this breed.

First, we tested the association between the mutant allele and a series of 28 traits that are routinely evaluated (including production, morphology, reproduction and health) as well as three coat color phenotypes. A strong association was found only with two udder traits, i.e. front teat distance and teat orientation, with an unfavorable effect of the mutant allele. Some putative effects of lower magnitude were also observed on fat and protein contents (Table 6). None of these effects can explain the high frequency of the mutant allele.

**Table 6:** Results of association studies between the *RP1* frameshift mutation and 31 traits routinely evaluated for 11,986 Normande cows

Traits	Effect	Standard error	p value (Bonferroni)
Front teat distance	−0.106	0.021	1.2E−05
Teat orientation	−0.076	0.019	2.4E−03
Milk protein content	−0.069	0.019	9.5E−03
Milk fat content	−0.132	0.037	9.9E−03

Only significant results after Bonferroni correction are presented. Details on the 31 different traits studied are presented in “Methods” section. Front teat distance and teat orientation are scored from 1 to 9. Milk protein and fat content are expressed in g/L

**Fig. 5:**
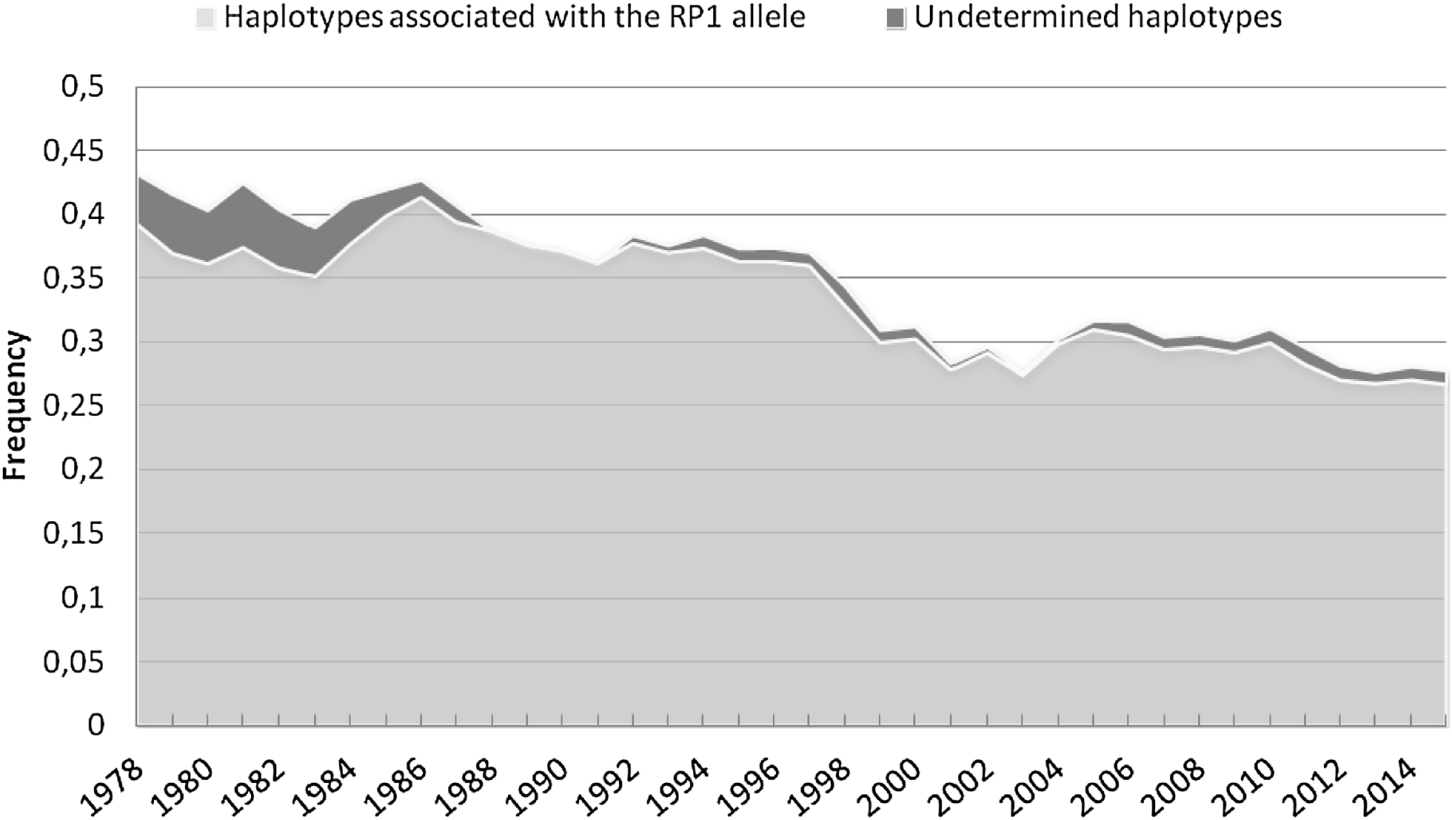
Changes in the frequency of the *RP1* frameshift allele within the AI Normande bulls population. Frequencies were calculated using a haplotype test approach for sliding windows of 7 years for AI bulls born from 1970 to 2015. Undetermined haplotypes correspond to haplotypes which were observed in both Fs/Fs and Wt/Wt animals genotyped for the *RP1* frameshift mutation with the Illumina EuroG10K SNP chip (see “Methods” section)

Second, using Illumina EuroG10K SNP genotyping data or phased Illumina BovineSNP50 haplotypes (see “Methods” section), we estimated the allelic frequencies over the last 40 years within the AI bull population (Fig. 5). Interestingly, the frequency of the *RP1* frameshift mutation showed a progressive decrease (from 40 to 27 %) during this period. Thus, the increase in frequency of the mutant allele in the Normande breed is more ancient and most probably results from a founder effect that was favored by the advent of AI in the 1950s. Because of the late onset of the defect and because the dams of the future AI bulls are primarily selected among young cows to reduce generation intervals and increase the annual genetic gain, it is unlikely that the decrease in allelic frequency is caused by selection against blindness. A possible explanation of this negative trend is the association of the mutant allele with udder morphology and the strong selection on this trait in the last 50 years. Indeed, the original udder morphology of Normande cows was not adapted to machine milking and was gradually improved over time through drastic selection. While this *RP1* mutation has very limited economic impact, it has major implications in terms of animal welfare and human safety. Indeed, with a frequency of 27 % in 2015, about one in every 14 Normande animals will become progressively blind and be subject to increased stress and fear, as we observed during sampling. This also means that each farmer possesses more than one homozygous carrier and has an increased risk of being injured by a startled animal. The identification of this mutation and its incorporation into the EuroG10K SNP chip used for genomic selection provide the basis for its active counter-selection.

## Conclusions

In this work, we have drawn a list of putative deleterious mutations which are not rare (frequency higher than 5 %) in at least one of 15 investigated European bovine breeds. We showed that these variants, which likely represent a substantial part of the genetic load of domestication in cattle, did not accumulate randomly. Our results reveal that mutations in genes, which are involved in cognition or sensorial functions for which little or no selection pressure exists in domesticated animals, were more tolerated than mutations that affect other systems, which are of primary importance for production, reproduction and survival. Among these variants, we described an ancestral frameshift mutation in *RP1* which segregates in numerous breeds and causes progressive retinal degeneration. To our knowledge, this is the most ancient and widespread mutation causing a recessive genetic defect in cattle reported to date. This example illustrates that our approach can help to unravel variants that are yet to be discovered and are the cause of unselected but debilitating phenotypes in domestic animals. We are confident that the phenotypic characterization of a number of the variants reported here will offer interesting results in the near future.

## Additional files

10.1186/s12711-016-0232-y Details on animals used in the phenotypic characterization of the *RP1* frameshift. This table provides details on the animals used for each analysis in this study. AAN: Aberdeen-Angus, BBB: Belgian Blue, BBR: Beef Booster Composite, CHA: Charolais, GVH: Gelbvieh, HOL: Holstein, NOR: Normande, NOR*HOL: Normande*Holstein crossbred RAN: Red Angus, RDP: Maine-Anjou.
10.1186/s12711-016-0232-y Details on non-rare putative deleterious variants selected in 15 breeds from the 1000 bull genomes run4 dataset. This file contains the list of the 2489 variants selected in this study. For each of them, we indicate: (i) the frequency in each breed, (ii) the functional consequence on the protein, and (iii) the genetic syndromes associated with mutations within the same gene in human (Online Mendelian Inheritance in Man, OMIM; http://www.omim.org) and mouse (Mammalian Phenotypes; http://www.informatics.jax.org). AAN: Aberdeen-Angus, BBR: Beef Booster Composite, BSW: Brown Swiss, CHA: Charolais, GVH: Gelbvieh, HER: Hereford, HOL: Holstein, JER: Jersey, LIM: Limousine, MON: Montbéliarde, NOR: Normande, RAN: Red Angus, RDCF: Finnish Red, RDCS: Swedish Red, and SIM: Simmental.
10.1186/s12711-016-0232-y Additional information on the results of the gene enrichment analysis performed with Ingenuity Pathway Analysis.  This file contains five tables providing additional details on the results of the gene enrichment analysis: a summary of the significant diseases and disorders annotations (Table S3), a summary of significant physiological system development and function annotations (Table S4), a summary of significant molecular and cellular functions annotations (Table S5), the key-word attribution to each significant functional annotation conserved for the analysis (Table S6) and the significant canonical pathways (Table S7).
